# Antibiotic Prophylaxis of Infective Endocarditis

**DOI:** 10.1007/s11908-017-0564-y

**Published:** 2017-02-23

**Authors:** Martin H. Thornhill, Mark Dayer, Peter B. Lockhart, Bernard Prendergast

**Affiliations:** 1 Department of Oral & Maxillofacial Medicine and Surgery, University of Sheffield School of Clinical Dentistry, Claremont Crescent, Sheffield, S10 2TA UK; 2 Department of Oral Medicine, Carolinas Medical Center, Charlotte, NC USA; 3 Taunton and Somerset NHS Trust, Taunton, UK; 4 Guy’s and St Thomas’ Hospitals, London, UK

**Keywords:** Infective endocarditis, Antibiotic prophylaxis, Endocardium infection

## Abstract

Links between infective endocarditis (IE) and dental and other invasive procedures were first identified in the 1920s, and the use of antibiotic prophylaxis (AP) to prevent IE was first recommended by the American Heart Association in 1955. Recognising the weak evidence to support this practice and the wider risks of anaphylaxis and antibiotic resistance, guidelines in the USA and Europe have been rationalised in the last decade with restriction of AP to those patients perceived to be at the highest risk. In the UK, the National Institute for Health and Care Excellence controversially recommended the complete cessation of AP for all invasive procedures in 2008 and subsequent epidemiological studies have suggested a significant increase in cases above the baseline trend. AP appears to be safe and is likely to be cost-effective. Until further data are available, we recommend continued adherence to US and European guidelines.

## Introduction

Infective endocarditis (IE) is an infection of the endocardium (particularly the valve leaflets) with a yearly incidence of 3–10 per 100,000 [1–3] and is characterised by the development of infected heart valve vegetations. Prognosis is poor with an in-hospital mortality of 15-20%, rising to approximately 30% at 1 year [1–3]. Prolonged high-dose intravenous antibiotics are the mainstay of treatment, but surgery (valve repair or replacement) is required in 40–50% of cases [1–3]. Morbidity is high in those who survive, with a significant risk of re-infection or relapse, as well as progressive deterioration in valve function leading to heart failure and the need for further medical and surgical intervention [1–3]. With such high mortality and morbidity, prevention strategies have always been a priority.

## Historical Background

The first suggestion that IE might be caused by microorganisms came from Winge in 1870 [4]. Klebs [5] and others confirmed that IE was an infectious disease, and experiments by Rosenbach [6], Wyssokowitsch [7] and others in the late 1800s established that bacteria entering the circulation could colonise damaged heart valves. Osler identified the importance of fibrin and platelet deposition on damaged endocardium and the primary role of microorganisms in pathogenesis [8–10]. However, the concept that bacteria released into the circulation during invasive dental procedures might cause IE was first suggested by Lewis and Grant in 1923 [11] and confirmed in 1935 by Okell and Elliott [12] who demonstrated that 61% of patients following dental extraction had a positive blood culture for oral viridans group *Streptococci* and that oral viridans group *Streptococci* could be isolated from the vegetations of 40–45% of IE cases.

These observations occurred during a period when the focal infection hypothesis implicated oropharyngeal sepsis as the cause of many systemic diseases [13, 14] leading to the systematic removal of teeth and other tissues in an attempt to prevent conditions such as IE. Following a critical appraisal of the focal infection hypothesis by Reinmann and Havens in 1940, this practice gradually drew to a close [15, 16].

The antimicrobial effects of sulphonamides were first recognised in the 1930s. This was soon followed by the suggestion that their use as antibiotic prophylaxis (AP) could reduce the risk of IE in patients with rheumatic heart disease undergoing invasive dental procedures [17–19]. Hirsch et al. [20] subsequently demonstrated reduced streptococcal bacteraemia in a group receiving penicillin prophylaxis compared to controls, paving the way for the American Heart Association (AHA) to produce the first official guidelines on the use of AP in 1955 [21].

## Initial Guidelines on the Use of Antibiotic Prophylaxis to Prevent IE

The first AHA guidelines identified those with rheumatic or congenital heart disease as being at increased risk of IE, and “dental extraction and other dental manipulations which disturb the gums, the removal of tonsils and adenoids, the delivery of pregnant women, and operations on the gastrointestinal or urinary tracts” as procedures where AP was indicated. They recommended intramuscular penicillin (600,000 units of aqueous penicillin or 500,000 units of procaine penicillin in oil containing 2% aluminium monostearate) 30 min before dental procedures. An alternative, but less desirable, oral penicillin regimen was also described (250,000–500,000 units “one-half hour before each meal and at bedtime, beginning twenty-four hours prior to the operation and continuing for 5 days”, an extra dose of 250,000 units being desirable at the time of the procedure).

Over the next two decades, there were four further iterations of the AHA guidelines [22–25]. During this period, the recommendation that women should receive AP during childbirth was dropped. However, the emergence of valve replacement surgery during the 1960s and 1970s [26–29] brought recognition that these patients were at particularly high-risk of IE. As a result, the 1975 guidelines recommended that these patients receive an AP regimen consisting of intramuscular streptomycin (1 g) plus intramuscular penicillin (1,000,000 units of aqueous penicillin G or 600,000 units of procaine penicillin G), whilst other at-risk patients were recommended intramuscular penicillin alone or in combination with streptomycin. Importantly, the possibility that bacteraemia with oral organisms could occur in the absence of dental procedures and the consequent need to “maintain the highest level of oral health” were recognised for the first time.

## The Move Towards Single Oral Dose Antibiotic Prophylaxis Regimens

In 1982, the British Society for Antimicrobial Chemotherapy (BSAC) produced the first UK guidelines [30]. The main difference between these and the 1977 AHA guidelines was a shift away from complex parenteral or multi-dose oral AP regimens, as these had been associated with poor compliance by dentists [31]. Instead, a single 3 g oral dose of amoxicillin 1 hour before the procedure was recommended on the grounds of effectiveness and improved compliance. Furthermore, the same protocol was recommended for all patients at increased risk of IE, including those with prosthetic valves. Parenteral antibiotic regimens were only recommended for those undergoing a general anaesthetic. Subsequent AHA and ESC guidelines moved towards wider adoption of simple oral AP regimens and the adoption of clindamycin in preference to erythromycin for those allergic to penicillins.

Following an IE symposium in Lyon in 1993, an international group of experts reviewed all guidelines (French, German, Dutch, Scandinavian, UK, and US) and published proposals for European consensus [32]. They categorised patients at high risk of IE (those with prosthetic valves, congenital heart disease causing cyanosis and previous IE) or lower-risk (those with valvular heart disease, including mitral valve prolapse with regurgitation and bicuspid aortic valve), and provided a list of cardiac conditions not-at-risk for IE. They highlighted the need for AP prior to invasive dental procedures associated with gingival bleeding, tonsillectomy, adenoidectomy and some gastrointestinal and urological procedures but indicated there was little evidence to support a risk of IE with endotracheal intubation, fiberoptic procedures, colposcopy, vaginal hysterectomy or vaginal delivery.

The 1997 AHA guidelines divided patients into high-risk, moderate-risk and negligible-risk categories [33]. There was close agreement with the European Consensus guidelines [32], although surgically constructed systemic pulmonary shunts and conduits were added to the high-risk category. Dental and other procedures that should and should not be covered with AP were also more clearly defined. For the first time, the AHA guidelines recommended a single 2 g oral dose of amoxicillin as the preferred AP regimen for all patients at risk of IE undergoing dental, oral, respiratory tract or oesophageal procedures, with clindamycin 600 mg as a single oral dose 1 h before the procedure for those allergic to penicillin. These guidelines were broadly matched by the 2004 European Society of Cardiology (ESC) guidelines [34] so that most international guidelines were closely aligned.

## The Move to Restrict the Number of Individuals Who Should Receive Antibiotic Prophylaxis

In 2006, BSAC published new AP recommendations for the UK [35] and, supported by a Cochrane review [36], argued that there was no evidence to support the use of AP during invasive dental procedures. Nonetheless, they stopped short of recommending cessation of AP for invasive dental procedures and instead recommended it should only be given to those at high risk of IE and its complications (those with a previous history of IE, mechanical or biological prosthetic valves or a surgically constructed systemic or pulmonary shunt or conduit). However, for those undergoing a broad range of invasive gastrointestinal, genitourinary, gynaecological and respiratory procedures, they continued to recommend AP for those at both moderate and high risks of IE.

The suggestion that AP for invasive dental procedures should be restricted to those at highest risk caused outrage on the part of UK cardiologists [37, 38], and the topic was referred to the newly formed National Institute for Health and Care Excellence (NICE). In March 2008, the NICE revealed the outcome of its review, and to the disbelief and shock of many cardiologists and cardiothoracic surgeons, recommended the complete cessation (for all procedures and all patients) of AP to prevent IE [39]. For dentists, this recommendation simplified patient management, removed a serious cause for concern and was rapidly and widely adopted [40, 41]. The main reasons given by the NICE for this recommendation were the lack of evidence to support the effectiveness of AP [39] and the results of their health economic analysis that concluded AP was not cost-effective [42].

Whilst the NICE guideline review was underway, the AHA announced the outcome of their latest guideline review, in 2007 [43]. Like BSAC, they recommended that the use of AP should be restricted to those at the highest risk of IE or its complications (Table 1) and that AP should cease for those at moderate risk (e.g. those with native or rheumatic valve disease). Beyond the BSAC recommendations, they also advised that AP should cease for many non-dental invasive procedures (including all genitourinary and gastrointestinal procedures).

**Table 1:**
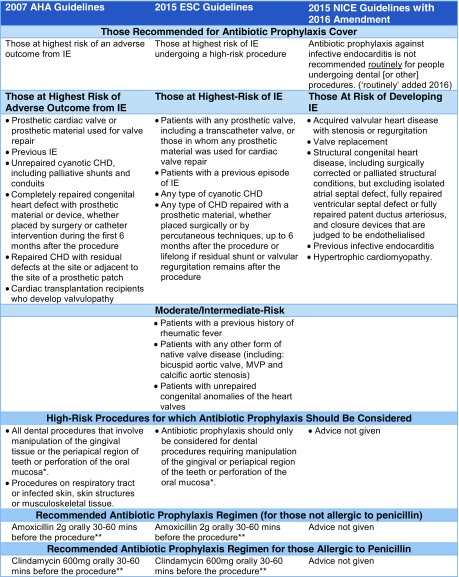
Current guidelines on antibiotic prophylaxis to prevent infective endocarditis (IE)

*CHD* congenital heart disease, *MVP* mitral valve prolapse, *ASD* atrial septal defect, *VSD* ventricular septal defect

^*^Excluding local anaesthetic injections through uninfected tissue (see original guidelines for all other exclusions)

^**^Please see original guidelines for children’s doses and parenteral and other alternative regimens

These recommendations were closely mirrored in the 2009 ESC guidelines that restricted AP to those at the highest risk of IE. The AHA or ESC guidelines have been widely adopted around the world (excluding Sweden who have adopted the UK NICE guidelines).

## The Impact of Changing Antibiotic Prophylaxis Guidelines

The best evidence for efficacy of AP would come from a randomised placebo controlled trial (RCT). Unfortunately, such a trial has never been performed and is unlikely in the foreseeable future due to cost and logistic reasons. Many hundreds of thousands of individuals at risk of IE would need to be randomised to placebo or prophylaxis to have sufficient statistical power to detect an effect [44]. A further barrier is the ethical concern of randomising individuals at risk of IE to placebo, particularly in countries where AP is the current standard of care [44].

Nevertheless, several recent observational studies have attempted to determine whether recent changes in AP guidelines have altered the incidence of IE. In 2012, Duval et al. reported three 1-year population-based studies from three French regions [45] assessing the impact of the 2002 French guidelines which recommended that AP should continue in those at high risk but was optional in those at lower risk [46]. Comparing two periods before the guideline change (1991 and 1999) with a period after (2008), they found no significant change in the incidence of IE (or oral streptococci IE). However, there were no prescribing data to demonstrate actual AP prescribing and the 2004 ESC guidelines may have influenced AP prescribing in France by 2008.

A number of studies have examined the impact of the 2007 AHA guidelines. Rogers and Schiller reported no increase in the number of IE admissions at a San Francisco Medical Center 9 months after the guideline change [47], and Desimone et al. examined Olmsted County data from 1999 to 2010 and concluded there was no increase in the incidence of viridans group streptococcal IE [48]. However, the small sample size and short follow-up period of both studies need consideration. Bor et al. used 1998–2009 data from the National Inpatient Sample to assess a broader cohort of patients but found no significant increase in the incidence of IE (overall or secondary to streptococcal infection) although the follow-up period was short. [49] Indeed, in a subsequent study of the same data set with 4 years follow-up, Pant et al. identified an increase in the incidence of streptococcal IE [50]. Although there are a number of limitations to this study, including the lack of any AP prescribing data [49], the authors speculated that the increase in streptococcal IE could result from decreased use of AP following the 2007 AHA guidelines. Another study by Pasquali et al. used the Pediatric Health Information Systems Database to study the effect of the 2007 guidelines on the incidence of IE between 2003 and 2010 in children age <18 years admitted to 37 US children’s hospitals and found no significant change in the incidence of total or streptococcal IE [51]. Similarly, Bikdeli et al. found no significant effect in a study of over 65 year olds with a principal or secondary diagnosis of IE from 1999 to 2010 using Medicare Inpatient Standard Analytic Files [52].

However, all of these studies are limited by one or more of the following shortcomings: small sample size, short follow-up period, sub-population studies with different risk factors or exposure to invasive dental procedures compared to the general population, use of secondary as well as primary IE diagnoses, difficulties in accurately identifying IE caused by oral viridans group streptococci and a lack of data on AP prescribing. Thus, it is very difficult to draw firm conclusions from any one or a combination of these studies [49].

In 2016, Mackie et al. used the Canadian Institute for Health Information Discharge Abstract Database to identify primary diagnoses of IE in Canada between 2002 and 2013 [53••]. Although there was no increase in the overall incidence of IE following the AHA guidelines, sub-analysis demonstrated that the declining incidence of streptococcal IE was reversed after 2007. A similar pattern was also noted in individuals at moderate risk of IE (the group for whom AP ceased in 2007) [54, 55].

The 2008 NICE guidelines recommended the complete cessation of AP in the UK where previously it had been recommended for those at moderate and high risk of IE. A 2011 study by Thornhill et al. of the 53 million population of England identified no significant increase in the incidence of IE in the subsequent 2 years despite a 78.6% fall in AP prescribing [41]. Definitive conclusions were not possible however owing to the short follow-up period and residual 21.4% level of AP prescribing [56, 57]. The study was therefore repeated with 5 years follow-up demonstrating a further decrease in AP prescribing (overall 88% fall) and a significant increase in the incidence of IE in the period following introduction of the NICE guidelines (*p* < 0.0001) which exceeded pre-existing trends [58••]. Thus, by March 2013 in England alone, there were an extra 419 IE cases per year than expected (*p* < 0.0001; 95% CI 117–743). These observational data do not prove that the fall in AP prescribing caused the increase in IE although a careful search for alternative causes has not identified a satisfactory explanation. Moreover, the study identified a significant increase in the incidence of IE in both high-risk (*p* < 0.001) and lower-risk (*p* = 0.01) groups, raising the possibility that AP may be of some benefit to those at moderate risk of IE.

## Recent Guideline Reviews

This spate of epidemiological evidence led the NICE to review its guidance at the same time as a scheduled review of ESC practice guidelines for the prevention, investigation and management of IE. Meanwhile, the AHA has announced approval for a review, but this it is not expected before 2018.

In September 2015, the NICE and the ESC announced their updated guidance having evaluated exactly the same evidence—the results could not have been more different. The NICE deemed that there was insufficient evidence to warrant any change to their existing guidance and continued to recommend against AP [59•]. In contrast, the ESC concluded that “the weight of evidence and opinion is in favour of the efficacy and usefulness of AP in preventing IE in those at high-risk”. And that “AP should be given before invasive dental procedures to all patients at high-risk of IE”. [60••]. Furthermore, the ESC guideline committee considered but rejected the 2008 NICE guidance not to recommend AP on account of (a) the remaining uncertainties regarding estimations of the risk of IE; (b) the worse prognosis of IE in high-risk patients (particularly those with prosthetic valves); and (c) the fact that high-risk patients account for a very small proportion of those previously covered by AP, thereby reducing the number exposed to any possible harmful adverse effects. The ESC recommended AP only for high-risk individuals undergoing high-risk invasive dental procedures and the AP regimen remained unchanged from 2009 [60••]. Its guidance therefore remains similar to that of the AHA (Table 1).

## How Can the ESC and NICE Differ So Much in Their Interpretation of the Same Evidence?

The ESC and AHA guideline committees consist of clinicians with relevant clinical expertise, including cardiologists, cardiothoracic surgeons, infectious disease experts and dentists. In both cases, the relevant professional bodies reviewed the guidance before submission to international peer reviewed journals where they were subjected to further external review. Both committees reviewed the available evidence (including animal and observational studies) before reaching their conclusions.

In contrast, the primary purpose of NICE is to evaluate the cost-effectiveness of drugs and technologies for use in the UK National Health Service. The AP review was performed by a 14-member standing committee that deals with a variety of different guidelines but has no particular expertise in IE. NICE review committees work to set protocols, largely designed for the evaluation of treatments for which RCT data are available. Animal studies are automatically excluded and even the best observational studies are deemed of “low quality” [59•].

Following their initial decision, the NICE came under considerable pressure from academics, cardiologists, dentists, patients and politicians to reconsider their view. As a consequence, their guidance was subtly changed in July 2016 to state that “AP against IE is not recommended routinely for people undergoing dental procedures”. The addition of the word “routinely” indicates that AP may be appropriate in individual cases [61•] and, in the absence of clear guidance concerning which individuals should receive AP, which dental procedures should be covered and what AP regimes should be used, places the responsibility for decisions concerning the use of AP with cardiologists and dentists. In this situation, it has been suggested by some that clinicians in the UK should follow recommendations based on the 2015 ESC guidelines [60••, 61•, 62].

## The Costs and Benefits of Antibiotic Prophylaxis

In the absence of RCT data, the benefits of AP are hard to quantify, although guideline committees implicitly acknowledge that there is a benefit in recommending AP for certain patient groups. *If* caused by the fall in AP prescribing, the increase in incidence of IE identified in the UK following introduction of the NICE guideline in 2008 suggests that 277 (95% CI 156–1217) prescriptions of AP are needed to prevent one case of IE and provides a reasonable estimate of AP effectiveness [58••]. The main potential problems with AP are (i) risk of adverse drug reactions, (ii) cost and (iii) risk of promoting antibiotic resistance.

A recent study [63••] found no recorded cases of death associated with amoxicillin AP (single 3 g oral dose) and a very low rate of non-fatal adverse reactions (22.6/million prescriptions). The incidence of adverse reactions with clindamycin AP (single 600 mg oral dose) was also low but higher than anticipated with 13 and 149 fatal and non-fatal adverse reactions/million prescriptions, respectively, nearly all of which related to *Clostridium difficile* infections. These data demonstrate a very high level of safety of amoxicillin AP in individuals with no history of allergy and suggest that an alternative to clindamycin (or no AP at all) should be considered for those who are allergic to penicillins. Unfortunately, these data were unavailable for the recent NICE and ESC guideline reviews but may be considered in future guideline reviews.

The cost of AP is modest [64] whereas the cost of treating IE is very high. However, the two most critical elements in evaluating the cost-effectiveness of AP are the incidence of adverse drug reactions and the efficacy of AP. Lack of cost-effectiveness was one of the reasons given by the NICE in 2008 for recommending the cessation of AP in the UK [39]. However, their cost-effectiveness analysis assumed little or no effectiveness for AP and used historical data (including long term parenteral penicillin use) which over-estimated the rate of adverse drug reactions [42, 65]. Perhaps not surprisingly, they concluded that AP was not cost-effective. In contrast, a recent health-economic study using contemporary efficacy and adverse reaction data found that AP was highly cost-effective (and even cost saving) [66].

Unfortunately, there are no data concerning the risk of inducing antibiotic resistance associated with AP. However, most concern relates to the use of lower dose therapeutic antibiotic regimens over several days and not to use of a single high dose of a bactericidal antibiotic, such as amoxicillin.

## Conclusions

The concept of AP to prevent IE has changed considerably since it was first formally introduced in the AHA guidelines of 1955 [21]. Dose and administration regimens have become simpler and shorter, and the number of individuals and procedures where AP is recommended has significantly reduced. Virtually all guideline committees around the world recommend AP for high-risk individuals undergoing high-risk invasive dental procedures (Table 1).

In the absence of RCT data to confirm the effectiveness of AP, observational studies are vital to determine the impact of guideline recommendations on the incidence of IE. Unfortunately, most observational studies are underpowered or have other methodological limitations. Most studies have shown little, if any, adverse impact of limiting AP to those at high risk of IE. However, some of the best designed and conducted studies suggest that complete cessation of AP may increase the incidence of IE [50, 53••, 54, 55, 58••]. More focussed and better-powered studies should eventually clarify this longstanding issue and provide better data concerning the risk of adverse drug reactions, cost-effectiveness of AP and the particular populations (if any) to whom AP should be targeted. At present, the stance currently adopted by most guideline committees limiting AP use to those at highest risk seems pragmatic and appropriate.

## References

[1] Cahill TJ, Prendergast BD (2015). Infective endocarditis. Lancet.

[2] Hoen B, Duval X (2013). Infective endocarditis. N Engl J Med.

[3] Murdoch DR, Corey GR, Hoen B, Miro JM, Fowler VG, Bayer AS (2009). Clinical presentation, etiology, and outcome of infective endocarditis in the 21st century: the International Collaboration on Endocarditis-Prospective Cohort Study. Arch Intern Med.

[4] Winge E (1870). Endocraditis (Mycosis endocardii). Nord Med Ark.

[5] Klebs E (1878). Weitere Beitrage zur Enststehungsgeschichte der endocarditis. Arch Exp Pathol Pharmakol.

[6] Rosenbach O (1878). Ueber artificielle Herzklappenfehler. Arch Exp Pathol Pharmakol.

[7] Wyssokowitsch V (1886). Beitrage zur Lehre von der Endocraditis. Arch Pathol Anat Phys.

[8] Osler W (1885). The Gulstonian lectures, on malignant endocarditis. Br Med J.

[9] Osler W (1885). The Gulstonian lectures, on malignant endocarditis. Br Med J.

[10] Osler W (1885). The Gulstonian lectures, on malignant endocarditis. Br Med J.

[11] Lewis T, Grant R (1923). Observations relating to subacute infective endocarditis. Heart.

[12] Okell CC, Elliott SD (1935). Bacteraemia and oral sepsis: with special reference to the aetiologu of subacute endocarditis. Lancet.

[13] Billings F (1914). Mouth infection as a source of systemic disease. JAMA.

[14] Billings F (1916). The principles involved in focal infection as related to systemic disease. JAMA.

[15] Focal infection. J Am Med Assoc. 1952;150:490–1. 10.1001/jama.1952.03680050056016 14955464

[16] Reimann HA, Havens WP (1940). Focal infection and systematic disease: a critical appraisal. The case against indiscriminate removal of teeth and tonsils. JAMA.

[17] Hupp JR (1993). Changing methods of preventing infective endocarditis following dental procedures: 1943 to 1993. J Oral Maxillofac Surg.

[18] Kolmer JA, Tuft L (1941). Clinical immunology, biotherapy and chemotherapy.

[19] Long PH, Bliss E (1939). Clinical use of sulfanilamide, sulfapyridine and allied compounds.

[20] Hirsch HL, Vivino JJ, Merril A, Dowling HF (1948). Effect of prophylactically administered penicillin on incidence of bacteremia following extraction of teeth. Arch Intern Med.

[21] Jones TD, Baumgartner L, Bellows MT, Breese BB, Kuttner AG, McCarty M (1955). Prevention of rheumatic fever and bacterial endocarditis through control of streptococcal infections. Circulation.

[22] American Heart Association (1960). Prevention of rheumatic fever and bacterial endocarditis through control of streptococcal infections. Circulation.

[23] American Heart Association Committee on Prevention of Rheumatic Fever and Bacterial Endocarditis (1965). Prevention of bacterial endocarditis. Circulation.

[24] American Heart Association (1972). Prevention of bacterial endocarditis. J Am Dent Assoc.

[25] Kaplan EL, Anthony BF, Bisno A, Drack D, Houser H, Millard DH (1977). Prevention of bacterial endocarditis. Circulation.

[26] Carpentier A (1971). The concept of bioprosthesis. Thoraxchir Vask Chir.

[27] Harken DE, Taylor WJ, Lefemine AA, Lunzer S, Low HB, Cohen ML (1962). Aortic valve replacement with a caged ball valve. Am J Cardiol.

[28] Ross DN (1962). Homograft replacement of the aortic valve. Lancet.

[29] Starr A, Edwards ML (1961). Mitral replacement: clinical experience with a ball-valve prosthesis. Ann Surg.

[30] Report of a working party of the British Society for Antimicrobial Chemotherapy The antibiotic prophylaxis of infective endocarditis. Lancet. 1982;2:1323–6. 6128610

[31] Brooks SL (1980). Survey of compliance with American Heart Association guidelines for prevention of bacterial endocarditis. J Am Dent Assoc.

[32] Leport C, Horstkotte D, Burckhardt D, Suppl B (1995). Antibiotic prophylaxis for infective endocarditis from an international group of experts towards a European consensus. Group of Experts of the International Society for Chemotherapy. Eur Heart J.

[33] Dajani AS, Taubert KA, Wilson W, Bolger AF, Bayer A, Ferrieri P (1997). Prevention of bacterial endocarditis. Recommendations by the American Heart Association. Circulation.

[34] Horstkotte D, Follath F, Gutschik E, Lengyel M, Oto A, Pavie A (2004). Guidelines on prevention, diagnosis and treatment of infective endocarditis executive summary; the task force on infective endocarditis of the European society of cardiology. Eur Heart J.

[35] Gould FK, Elliott TS, Foweraker J, Fulford M, Perry JD, Roberts GJ (2006). Guidelines for the prevention of endocarditis: report of the Working Party of the British Society for Antimicrobial Chemotherapy. J Antimicrob Chemother.

[36] Antibiotics for the prophylaxis of bacterial endocarditis in dentistry. [Review][Update of Cochrane Database Syst Rev. 2008;(4):CD003813; PMID: 18843649] Glenny AM; Oliver R; Roberts GJ; Hooper L; Worthington HV. 10.1002/14651858.CD003813.pub3 18843649

[37] Gibbs JL, Cowie M, Brooks N (2006). Defying explanation. Br Dent J.

[38] Ramsdale DR, Morrison L, Palmer MD, Fabri B (2006). Lethal consequences. Br Dent J.

[39] National Institute for Health and Care Excellence (NICE). Prophylaxis against infective endocarditis. Secondary prophylaxis against infective endocarditis. 2008;Volume:Pages.http://www.nice.org.uk/guidance/cg64. Accessed March 2008

[40] Dayer MJ, Chambers JB, Prendergast B, Sandoe JA, Thornhill MH (2013). NICE guidance on antibiotic prophylaxis to prevent infective endocarditis: a survey of clinicians’ attitudes. QJM.

[41] Thornhill MH, Dayer MJ, Forde JM, Corey GR, Chu VH, Couper DJ (2011). Impact of the NICE guideline recommending cessation of antibiotic prophylaxis for prevention of infective endocarditis: before and after study. BMJ.

[42] National Institute for Health and Care Excellence (NICE). Prophylaxis against infective endocarditis—Appendix 6. de novo economic analysis. Secondary prophylaxis against infective endocarditis—Appendix 6. de novo economic analysis. 2008;Volume:Pages.http://www.nice.org.uk/CG064. Accessed March 2008

[43] Wilson W, Taubert KA, Gewitz M, Lockhart PB, Baddour LM, Levison M (2007). Prevention of infective endocarditis: guidelines from the American Heart Association: a guideline from the American Heart Association Rheumatic Fever, Endocarditis, and Kawasaki Disease Committee, Council on Cardiovascular Disease in the Young, and the Council on Clinical Cardiology, Council on Cardiovascular Surgery and Anesthesia, and the Quality of Care and Outcomes Research Interdisciplinary Working Group. Circulation.

[44] Thornhill MH, Lockhart PB, Prendergast B, Chambers JB, Shanson D (2015). NICE and antibiotic prophylaxis to prevent endocarditis. Br Dent J.

[45] Duval X, Delahaye F, Alla F, Tattevin P, Obadia JF, Le Moing V (2012). Temporal trends in infective endocarditis in the context of prophylaxis guideline modifications: three successive population-based surveys. J Am Coll Cardiol.

[46] Danchin N, Duval X, Leport C (2005). Prophylaxis of infective endocarditis: French recommendations 2002. Heart.

[47] Rogers AM, Schiller NB (2008). Impact of the first nine months of revised infective endocarditis prophylaxis guidelines at a university hospital: so far so good. J Am Soc Echocardiogr.

[48] Desimone DC, Tleyjeh IM, de Sa DD C, Anavekar NS, Lahr BD, Sohail MR (2012). Incidence of infective endocarditis caused by viridans group streptococci before and after publication of the 2007 American Heart Association’s endocarditis prevention guidelines. Circulation.

[49] Dayer M, Thornhill M (2015). Antibiotic prophylaxis guidelines and infective endocarditis: cause for concern?. J Am Coll Cardiol.

[50] Pant S, Patel NJ, Deshmukh A, Golwala H, Patel N, Badheka A (2015). Trends in infective endocarditis incidence, microbiology, and valve replacement in the United States from 2000 to 2011. J Am Coll Cardiol.

[51] Pasquali SK, He X, Mohamad Z, McCrindle BW, Newburger JW, Li JS (2012). Trends in endocarditis hospitalizations at US children’s hospitals: impact of the 2007 American Heart Association Antibiotic Prophylaxis Guidelines. Am Heart J.

[52] Bikdeli B, Wang Y, Kim N, Desai MM, Quagliarello V, Krumholz HM (2013). Trends in hospitalization rates and outcomes of endocarditis among Medicare beneficiaries. J Am Coll Cardiol.

[53.••] Mackie AS, Liu W, Savu A, Marelli AJ and Kaul P. Infective endocarditis hospitalizations before and after the 2007 American Heart Association Prophylaxis Guidelines. Can J Cardiol. 2016; 32(8):942-8. **The largest observational study to date, using Canadian national data to characterise the impact of the 2007 AHA guideline restricting antibiotic prophylaxis to prevent infective endocarditis to those at the highest risk (see also the related correspondence, references 54 and 55).**

[54] Mackie AS, Liu W, Savu A, Marelli AJ and Kaul P. Reply to Letter From Thornhill *et al.*-Infective endocarditis hospitalizations before and after the 2007 American Heart Association Prophylaxis Guidelines. Can J Cardiol. 2016; 32(12):1578.e11. 10.1016/j.cjca.2016.04.002 27345608

[55] Thornhill MH, Dayer MJ, Jones S, Prendergast B, Baddour LM and Lockhart PB. The effect of antibiotic prophylaxis guidelines on incidence of infective endocarditis. Can J Cardiol. 2016; 32(12):1578.e9. 10.1016/j.cjca.2016.02.074 27160964

[56] Chambers JB, Shanson D, Hall R, Pepper J, Venn G, McGurk M (2011). Antibiotic prophylaxis of endocarditis: the rest of the world and NICE. J R Soc Med.

[57] Chambers JB, Shanson D, Venn G, Pepper J (2011). NICE v world on endocarditis prophylaxis. BMJ.

[58.••] Dayer MJ, Jones S, Prendergast B, Baddour LM, Lockhart PB, Thornhill MH (2015). Incidence of infective endocarditis in England, 2000–13: a secular trend, interrupted time-series analysis. Lancet.

[59.•] National Institute for Health and Care Excellence (NICE). Prophylaxis against infective endocarditis. Secondary Prophylaxis against infective endocarditis. 2015;Volume:Pages.http://www.nice.org.uk/guidance/cg64/chapter/Recommendations. Accessed 19-06-2015 **This represents the outcome of the 2015 review of the NICE guidelines in the UK.**

[60.••] Habib G, Lancellotti P, Antunes MJ, Bongiorni MG, Casalta JP, Del Zotti F (2015). 2015 ESC Guidelines for the management of infective endocarditis: The Task Force for the Management of Infective Endocarditis of the European Society of Cardiology (ESC)Endorsed by: European Association for Cardio-Thoracic Surgery (EACTS), the European Association of Nuclear Medicine (EANM). Eur Heart J.

[61.•] Thornhill MH, Dayer M, Lockhart PB, McGurk M, Shanson D, Prendergast B (2016). A change in the NICE guidelines on antibiotic prophylaxis. Br Dent J.

[62] Thornhill MH, Dayer M, Lockhart PB, McGurk M, Shanson D, Prendergast B (2016). Guidelines on prophylaxis to prevent endocarditis. Br Dent J.

[63.••] Thornhill MH, Dayer MJ, Prendergast B, Baddour LM, Jones S, Lockhart PB (2015). Incidence and nature of adverse reactions to antibiotics used as endocarditis prophylaxis. J Antimicrob Chemother.

[64] Lockhart PB, Blizzard J, Maslow AL, Brennan MT, Sasser H, Carew J (2013). Drug cost implications for antibiotic prophylaxis for dental procedures. Oral Surg Oral Med Oral Pathol Oral Radiol.

[65] Clemens JD, Ransohoff DF (1984). A quantitative assessment of pre-dental antibiotic prophylaxis for patients with mitral-valve prolapse. J Chronic Dis.

[66] Franklin M, Wailoo A, Dayer M, Jones S, Prendergast B, Baddour LM, Lockhart PB and Thornhill MH. The cost-effectiveness of antibiotic prophylaxis for patients at risk of infective endocarditis. Circ. 2016;134:1568–78. 10.1161/CIRCULATIONAHA.116.022047 PMC5106088 27840334

